# Supplementary material for: Perturbed neurochemical and microstructural organization in a mouse model of prenatal opioid exposure: A multi-modal magnetic resonance study
Source: PLoS One. 2023 Jul 20;18(7):e0282756. doi: 10.1371/journal.pone.0282756 (PMC10358947; doi:10.1371/journal.pone.0282756)
Supplement: S1 File — (DOCX) [file pone.0282756.s001.docx]

# **Supporting Information**

**S1 Figure:** Partial correlation between Glu and NAA. Glu was significantly correlated with NAA in PSE offspring.

(PSE: R^2^=0.65; p<0.001)

(PME: R^2^=0.02; p=0.65)

**S1 Table:** Group comparison of localized (MRS voxel based) diffusion microstructural metrices in right dorsal striatum between PSE (n=7) and PME offspring

| **Localized microstructural indices** | **PSE (mean±SD)** | **PME (mean±SD)** | **p-value** |
| --- | --- | --- | --- |
| FA | 0.20±0.02 | 0.22±0.01 | 0.06 |
| MD (mm^2^/s) | 4.7x10-4±2.1x10-5 | 4.7x10-4±1.8x10-5 | 0.81 |
| ODI | 0.44±0.05 | 0.42±0.04 | 0.53 |
| VF_IC_ | 0.61±0.07 | 0.61±0.05 | 0.99 |
| T1(ms) | 1512.35±180.76 | 1652.52±26.47 | 0.06 |

**S2 Table:** Partial correlation between neurometabolite concentrations and diffusion microstructural metrices (ROI: right dorsal striatum, PSE+PME)

| **Metabolites**  **[µmol/g]** | **FA** | | **MD** | | **ODI** | | **VF_IC_** | |
| --- | --- | --- | --- | --- | --- | --- | --- | --- |
|  | r | P value | r | P value | r | P value | r | P value |
| NAA | -0.533 | 0.06 | 0.393 | 0.184 | -0.025 | 0.935 | -0.238 | 0.433 |
| Tau | -0.581 | **0.037** | 0.241 | 0.427 | -0.068 | 0.826 | -0.330 | 0.271 |
| GSH | -0.333 | 0.266 | 0.132 | 0.668 | 0.013 | 0.968 | -0.020 | 0.949 |
| tCr | -0.465 | 0.110 | 0.229 | 0.452 | -0.006 | 0.986 | -0.210 | 0.492 |
| Glu | -0.582 | **0.037** | -0.036 | 0.908 | 0.252 | 0.406 | -0.043 | 0.888 |
| Gln | -0.308 | 0.306 | 0.479 | 0.098 | -0.241 | 0.427 | -0.340 | 0.255 |
| GABA | -0.135 | 0.659 | 0.300 | 0.320 | -2.121 | 0.487 | -0.123 | 0.689 |
| Ins | -0.290 | 0.337 | -0.106 | 0.731 | 0.241 | 0.427 | 0.027 | 0.929 |
| tCh | 0.100 | 0.746 | -0.177 | 0.564 | 0.029 | 0.925 | 0.157 | 0.608 |

**S3 Table:** Partial correlation between neurometabolite concentrations and diffusion microstructural metrices (ROI: right dorsal striatum, PSE)

| **Metabolites**  **[µmol/g]** | **FA** | | **MD** | | **ODI** | | **VF_IC_** | |
| --- | --- | --- | --- | --- | --- | --- | --- | --- |
|  | r | P value | r | P value | r | P value | r | P value |
| NAA | -0.705 | 0.118 | 0.602 | 0.206 | -0.341 | 0.508 | -0.517 | 0.293 |
| Tau | -0.495 | 0.318 | 0.638 | 0.173 | -0.535 | 0.275 | -0.547 | 0.261 |
| GSH | -0.063 | 0.906 | 0.284 | 0.586 | -0.437 | 0.386 | -0.244 | 0.641 |
| tCr | -0.418 | 0.409 | 0.584 | 0.224 | -0.531 | 0.279 | -0.503 | 0.309 |
| Glu | -0.611 | 0.198 | 0.503 | 0.309 | -0.360 | 0.484 | -0.427 | 0.398 |
| Gln | -0.351 | 0.495 | 0.622 | 0.187 | -0.555 | 0.253 | -0.544 | 0.264 |
| GABA | -0.678 | 0.139 | 0.143 | 0.786 | 0.036 | 0.946 | 0.048 | 0.928 |
| Ins | -0.650 | 0.163 | 0.075 | 0.888 | 0.108 | 0.839 | -0.119 | 0.822 |
| tCh | 0.039 | 0.941 | -0.103 | 0.846 | -0.233 | 0.656 | 0.093 | 0.861 |
